# 多孔有机骨架材料对顺式二醇化合物富集分离的研究进展

**DOI:** 10.3724/SP.J.1123.2022.04024

**Published:** 2022-11-08

**Authors:** An ZHANG, Juan ZHANG

**Affiliations:** 武汉工程大学化学与环境工程学院, 石油和化工行业生物质环境与能源新材料重点实验室, 湖北省新型反应器与绿色化学工艺重点实验室, 湖北 武汉 430205; School of Chemistry and Environmental Engineering, Wuhan Institute of Technology, Key Laboratory of Novel Biomass-Based Environmental and Energy Materials in Petroleum and Chemical Industry, Hubei Key Laboratory of Novel Reactor and Green Chemical Technology, Wuhan 430205, China

**Keywords:** 金属有机骨架, 共价有机骨架, 顺式二醇化合物, 选择性富集, 综述, metal organic frameworks (MOFs), covalent organic frameworks (COFs), *cis*-diol-containing compounds, selective enrichment, review

## Abstract

基于在碱性环境下硼酸能与顺式二醇化合物可逆共价结合形成稳定的五元或六元环酯,而在酸性环境下环酯开环释放顺式二醇化合物这一特性,设计合成高效、高选择性、高富集性能的硼亲和材料的研究备受关注。近年来,许多研究工作者合成了各种类型的硼亲和材料,应用于高选择性富集顺式二醇化合物。金属有机骨架(MOFs)和共价有机骨架(COFs)由于具有孔径可调、高孔隙率、高比表面积、骨架结构可调和化学及热稳定性良好等特点,被广泛应用于色谱分离和样品前处理领域。为赋予MOFs和COFs材料对顺式二醇化合物的富集选择性,各种不同结构和不同种类的硼酸修饰的MOFs和COFs被合成出来。该综述主要是对近几年来80余篇源于科学引文索引关于硼酸功能化MOFs和COFs的种类、合成方法及其应用文章的总结,包括“金属配体-片段共组装”“合成后修饰”和“自下而上”的硼酸功能化多孔材料的修饰策略,以及硼酸功能化MOFs和COFs的种类,介绍了其在化学分析和生物分析领域的发展概况和应用前景,客观评价了硼酸功能化MOFs和COFs的区别和优缺点。该文旨在让研究人员能够充分了解近几年硼酸功能化多孔有机骨架材料的研究现状、掌握合成思路和方法,为其应用提供一定的理论指导和技术支撑,为加快硼酸功能化多孔有机骨架材料的商业化脚步贡献绵薄之力。

## Abstract (en)

The design and synthesis of boronate affinity materials that show high efficiency, high selectivity, and high enrichment performance have gained significant attention. The principle of boronate affinity relies on the reversible covalent reactions, including the formation of stable five-membered or six-membered cyclic esters with *cis*-diol-containing compounds in alkaline aqueous media and dissociation of cyclic esters in an acidic surrounding to release *cis*-diol-containing compounds. Recently, various boronate affinity materials have been synthesized and utilized for selective enrichment of these compounds. Metal organic frameworks (MOFs) and covalent organic frameworks (COFs) have been widely used in chromatographic separation and sample pretreatment because of their adjustable pore size, high porosity, high specific surface area, tunable skeleton structure, and favorable chemical and thermal stability. To promote the enrichment selectivity of MOFs and COFs for *cis*-diol-containing compounds, boronic acid-functionalized MOFs and COFs with various structures and categories have been synthesized. This review summarizes more than 80 investigations into the categories, synthetic strategies, and applications of boronic acid-functionalized MOFs and COFs from the Science Citation Index. These synthesis methods include metal ligand-fragment co-assembly, post-synthetic modification, and bottom-up modification of boronic acid-functionalized porous materials. Although two modification strategies (post-synthetic and metal ligand-fragment co-assembly) have been introduced for the preparation of boronic acid-functionalized MOFs, the latter is more commonly adopted as it improves the enrichment selectivity and enrichment efficiency of MOFs. The common limitations of MOFs such as aggregation and aperture issues were also resolved. Boron affinity MOFs possessing favorable properties according to the characteristics of *cis*-diol-containing compounds, have also been synthesized. Furthermore, to facilitate enrichment and separation, many boronic acid-functionalized magnetic material MOFs have been developed for the enrichment and analysis of *cis*-diol-containing compounds. Additionally, the luminescent properties of Ln-MOFs have been used in combination with boronic acid affinity for the enrichment, separation, and subsequent detection of *cis*-diol-containing compounds. Post-synthetic modification and the bottom-up strategy are the primary methods for the preparation of boronic acid-functionalized COFs. Boronic acid-functionalized COFs are less investigated than boronic acid-functionalized MOFs, likely due to the greater complexity of COF synthesis. This work aims to summarize the research advances, synthesis ideas, and synthesis methods related to boric acid-functionalized porous organic frameworks, which will provide theoretical guidance and technical support for its applications while accelerating the commercialization of such organic frameworks.

近年来,许多顺式二醇化合物在生物、临床和工业领域具有重要的研究意义。例如,多巴胺^[1⇓-3]^、糖和糖肽^[4]^是当前代谢组学和蛋白质组学研究的重要目标;从污水中去除具有高毒性、致癌性、高需氧量和低生物降解性的邻苯二酚,对自然环境的修复具有重要意义^[5⇓-7]^。硼酸亲和色谱已被证明是选择性富集含顺式二醇分子的有力工具^[8⇓-10]^。硼酸亲和材料的分子相互作用原理依赖于硼酸配体与顺式二醇化合物之间的可逆共价反应。简而言之,硼酸在碱性条件下可以与顺式二醇反应生成五元或六元环酯。然而,当环境变为酸性时,环酯就会解离释放出顺式二醇化合物以实现选择性富集分离。研究工作者利用硼亲和色谱原理,设计合成了各种硼酸功能化材料用于顺式二醇化合物的富集分析,其中包括:介孔二氧化硅^[11⇓⇓-14]^、整体柱^[15]^、聚合物^[16]^和磁性纳米复合材料^[9]^等。近年来,在关于硼酸功能化材料的合成研究中,研究人员一直致力于提高硼酸官能团的接枝密度和硼酸亲和材料的稳定性。

共价有机骨架(COFs)和金属有机骨架(MOFs)由于具有丰富的孔径、优异的孔隙率、较高的比表面积和可调的骨架结构等特点,已被广泛用于吸附与分离领域。近几年,我们课题组在MOFs和COFs材料的设计合成及其在色谱分离、固相微萃取、磁性固相萃取应用等方面开展了大量的工作^[17⇓⇓⇓⇓⇓-23]^,设计合成了COF-1作为固定相用于毛细管电色谱分离^[17]^、氟化石墨烯和ZIF-8复合吸附剂用于水和食品中有机农药富集分离分析^[18]^、卟啉基COFs磁性吸附剂^[20]^及MOFs和COFs复合材料吸附剂等用于抗生素残留的富集分离和检测^[21,22]^、硼酸掺杂的磁性卟啉MOFs用于顺式二醇化合物如核苷的高选择性富集分离和检测^[23]^。越来越多的科研团队也利用MOFs和COFs材料的优势,设计合成了许多硼酸功能化的MOFs和COFs,并将它们运用于不同种类的顺式二醇化合物的吸附与分离的研究中。本文归纳整理了近年来部分硼酸功能化MOFs和COFs作用于顺式二醇化合物的文献记载,并在此基础上,结合相关研究情况,概述了硼酸功能化MOFs和COFs材料的相关应用及其未来的发展应用前景。

## 1 硼酸功能化MOFs

MOFs是一类由金属离子和有机连接体组成的周期性多孔晶体材料^[24]^。多项研究表明,MOFs具有独特的物理化学特性,包括多种拓扑结构、优异的孔隙度、可调节的孔径和骨架结构,因此在吸附与分离领域具有良好的应用前景^[25⇓-27]^。此外,通过含有特定官能团的配体,设计合成功能化MOFs以拓展其应用已成为科研工作者的研究重点。将原配体与目标官能团片段共同组装成MOFs,既保留了母体MOFs的结构^[28]^,又能改善目标官能团的部分缺点。近年来,具有硼酸官能团的配体已被成功组装,得到的功能化MOFs对顺式二醇化合物具有较好的选择性富集性能,大量的硼酸活性位点存在于吸附剂中,有效地提高了吸附剂的吸附容量和富集分离选择性^[29,30]^。如图1所示,本课题组近期也开展了硼亲和MOFs磁性材料的研究工作,采用双配体策略通过一步水热法合成了硼酸掺杂的卟啉MOFs磁性吸附剂用于核苷的磁性固相萃取,利用卟啉环的富氮骨架、卟啉环类似“井”的结构及接枝的硼酸官能团实现顺式二醇化合物的高选择性富集,并将建立的方法用于人尿液中核苷的高选择性富集分离分析^[23]^。所制备的磁性吸附剂由于比表面积较低,因此富集容量有待提高,后期我们将设计合成硼酸接枝密度高的亲水型多孔硼亲和材料,在减少非特异性吸附的同时,提高富集选择性和富集容量。

**图1:**
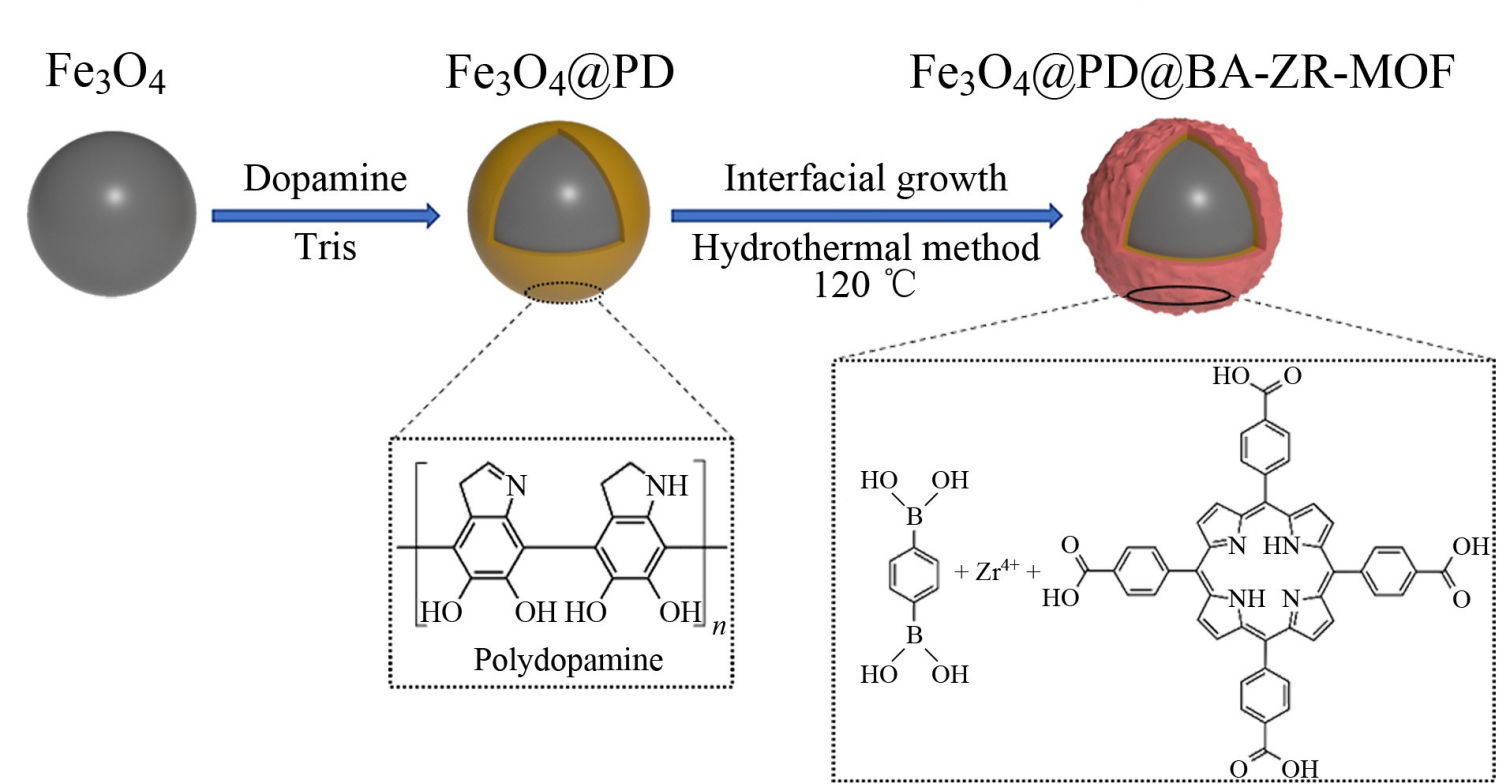
Fe_3_O_4_@PD@BA-Zr-MOF的制备示意图^[23]^

### 1.1 硼酸功能化MOFs的合成方法

#### 1.1.1 “金属配体-片段共组装”策略

金属配体-片段共组装策略(MLFC)^[31⇓-33]^是一种将介孔引入MOFs的极好方法,与传统的前功能化和后功能化方法相比,MLFC策略是将硼酸官能团原位生长在MOFs上,而不是通过减少孔体积来实现,这提高了MOFs的吸附效率,因此被广泛运用于硼酸功能化MOFs材料的合成中。然而目前合成的大多数MOFs分散性较差,聚集在一起使得硼酸官能团的吸附效率被极大地削弱,因此,研究人员通常会引入其他材料作为载体^[34]^。沸石咪唑骨架(ZIF)是MOFs的一个亚类,可被用作支撑其他材料的基体。以其作为基体的纳米材料具有合成条件简单、热稳定性好、化学稳定性高、孔容大等特点。Guo等^[35]^以ZIF-67为载体,通过多巴胺(DA)的自聚合能力,使其在表面形成聚多巴胺(PDA)层,采用MLFC策略以四氯化锆为反应前驱体,对苯二甲酸(TPA)、3-羧基苯基硼酸(3-CPBA)为双配体制备得到复合吸附剂(ZIF-67@PDA@BA-Zr-MOFs),并且将其成功应用于富集花生壳样品中的木犀草素(LTL)(见图2),为从复杂样品基质中快速提取和分离LTL提供了一个理想的方法。

**图2:**
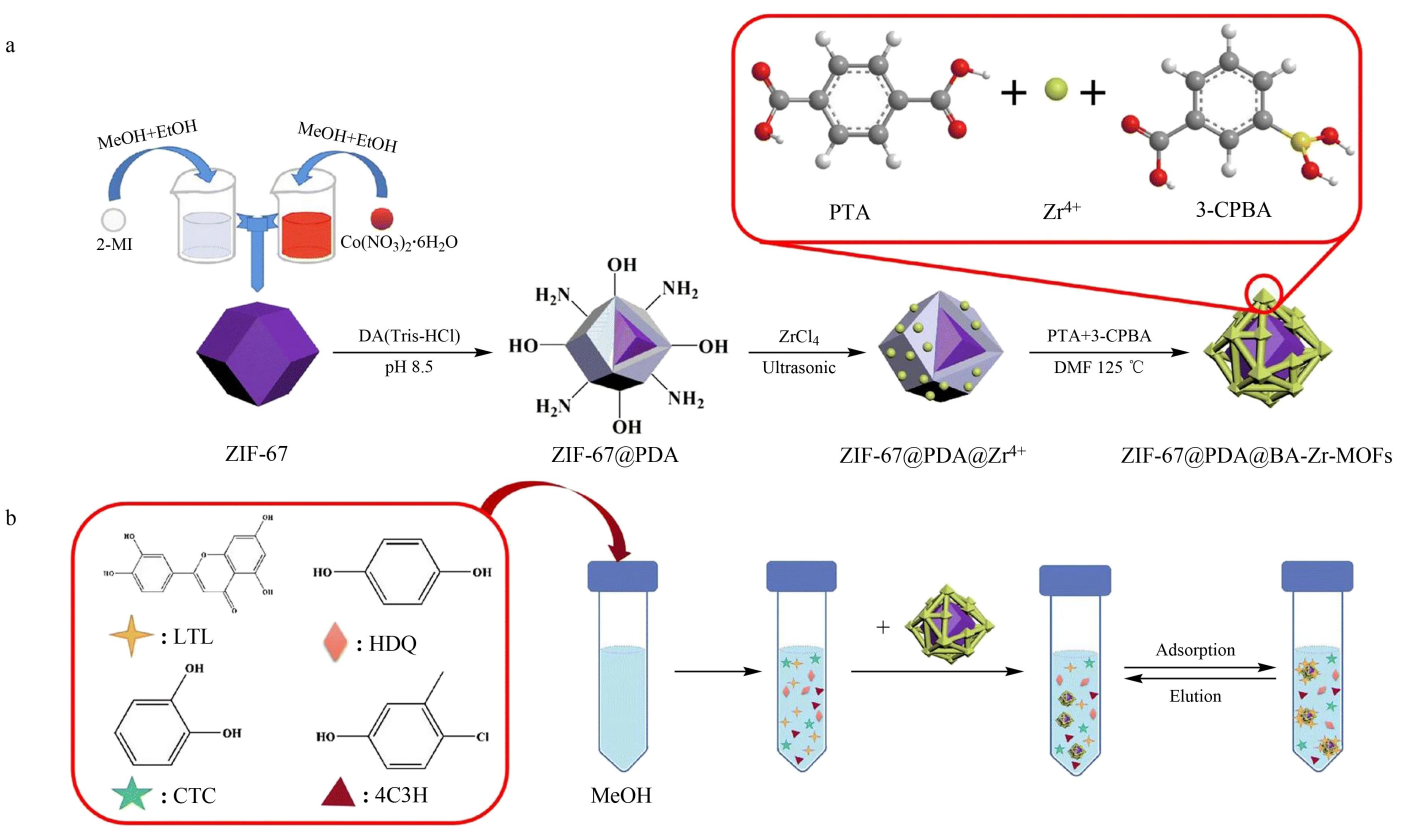
(a)ZIF-67@PDA@BA-Zr-MOFs的制备示意图和(b)固相萃取流程图^[35]^

Pankajakshan和Mandal^[36]^通过MLFC策略,将5-硼基异苯二甲酸(BIA)与三聚氰胺酸(BTC)配体按物质的量比分别为1:1、1:2、1:3、1:4、1:5和1:6,合成6组不同MOFs,测试了它们对水介质中各种顺式二醇化合物的结合能力,并且证实了随着金属有机骨架内硼酸含量的增加,其对顺式二醇吸附效率逐渐增加,在水中对各类糖的吸附能力大小顺序为半乳糖>葡萄糖>甘露糖>木糖。Zhu等^[28]^也是通过MLFC策略,将5-硼苯-1,3-二羧酸(BBDC)作为配体片段引入功能成分,制得新型MIL-100-B MOFs,实现对顺式二醇生物分子(CDBs)的高选择性捕获。该材料的比表面积较大,并且在较宽的pH范围内表现出优异的化学稳定性,使其在CDBs纯化、传感和分离等领域表现出巨大应用潜力。Chen等^[37]^设计了一种硼酸修饰的Cr基MIL-100纳米反应器B-D-MIL-100(见图3),并用于碳水化合物的快速分离和标记。He等^[38]^使用MIL-100(Fe)作为基本骨架(见图4),引入Fe^3+^作为金属中心,1,3,5-苯三羧酸作为主要有机配体,以5-硼苯-1,3-二羧酸为配体片段,在MIL-100空腔中引入活性硼酸,制备了MIL-100-B(硼酸功能化的MIL-100),用于捕获生物样品中的儿茶酚胺。MLFC方法能减少堵塞金属有机骨架的孔隙,这是相比合成后改性(PSM)策略的一个显著优势。

**图3:**
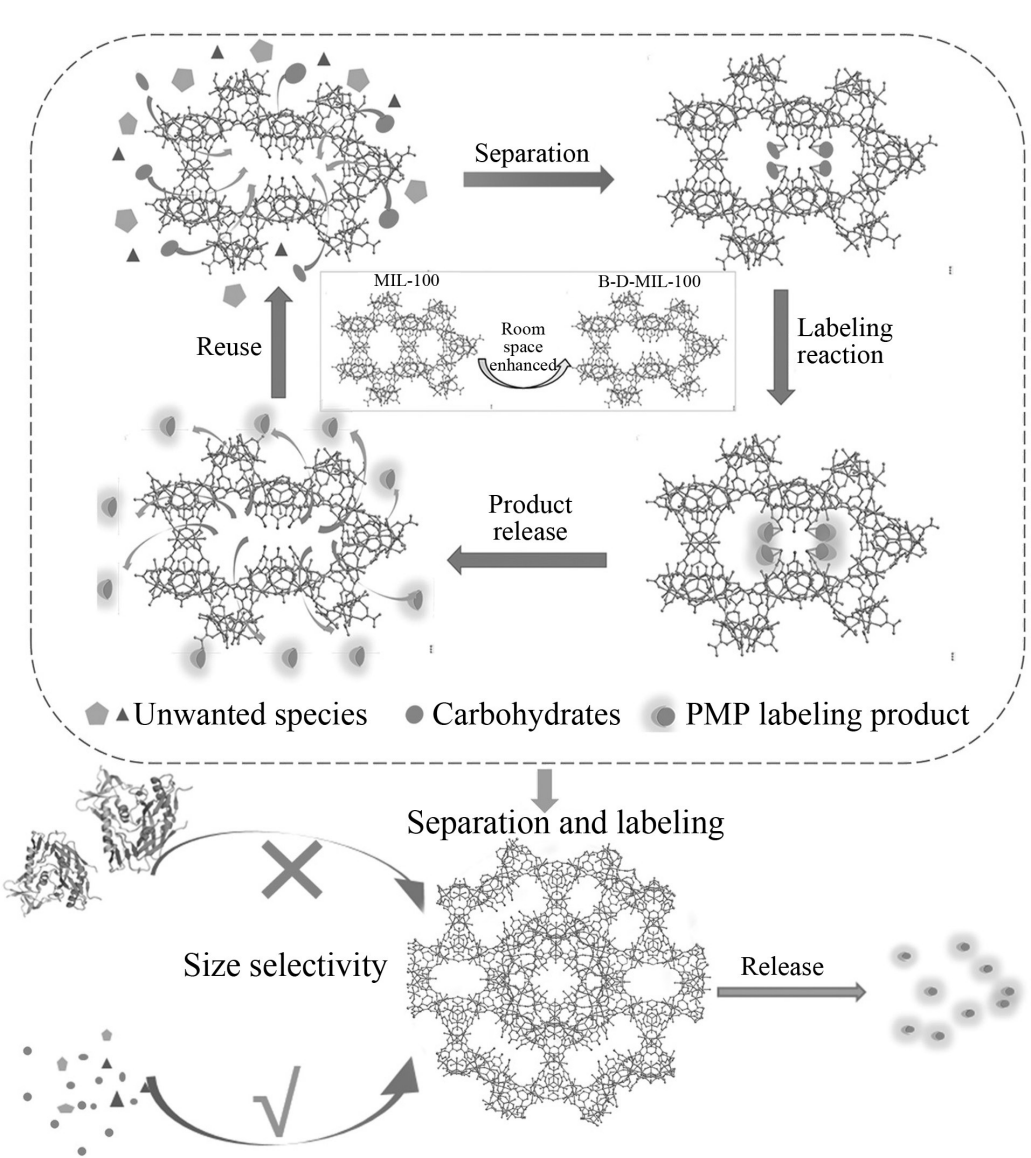
碳水化合物在硼酸修饰的缺陷金属有机骨架纳米反应器中的分离和标记^[37]^

**图4:**
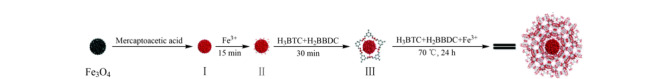
MG@MIL-100-B复合材料的合成方案^[38]^

在有机配体中,芳香或共轭*π*基团容易被激发并在辐射下发光^[39,40]^。但是,当UiO-66-NH_2_@B(OH)_2_的硼酸官能团与唾液酸甘油侧链的二醇基团结合时,UiO-66-NH_2_@B(OH)_2_的荧光强度会降低甚至猝灭。利用此特性,Yu等^[41]^以2-氨基对苯二甲酸(BDC-NH_2_)和4-羧基苯基硼酸(CPB)为有机配体,在UiO-66-NH_2_框架中引入硼酸官能团,用一步溶剂热法制备UiO-66-NH_2_@B(OH)_2_,此材料可以选择性识别和检测唾液酸(SA),因此该材料可被开发用于唾液酸相关疾病的临床诊断。

#### 1.1.2 “合成后修饰”策略

大多数报道的MOFs的孔径小于2 nm^[42]^,降低了扩散速率,阻碍了大分子进入内孔,影响了MOFs中吸附位点的利用^[43]^。通过调节材料孔径得到的分层多孔金属有机框架(HP-MOFs)可以有效地解决这些问题,HP-MOFs同时具有微孔和中孔,其中微孔具有较高的比表面积,中孔可以吸附较大的分子,因此,HP-MOFs进一步扩大了MOFs的应用^[44]^。扩大孔径的传统方法是使用细长的有机配体^[45]^。然而,有机配体的延长会破坏MOFs的稳定性,使其难以形成晶体结构。

Tan等^[46]^利用Zn基MOFs和Zr基MOFs对酸的稳定性上的差异,在双金属MOFs(Zn/Zr-MOFs)中对Zn基MOFs进行酸蚀,得到了分级孔,并对其进行硼酸功能化修饰(见图5),制备了一种新型的硼酸亲和分层孔MOFs(BA-HP-UiO-66),用于富集替考拉宁。研究表明所制备的层级硼酸亲和MOFs对大分子的顺式二醇化合物具有良好的吸附性能。BA-HP-UiO-66的平均孔径从原始UiO-66-NH_2_的0.85 nm增大到2.40~3.83 nm,并且,得到的BA-HP-UiO-66具有较高的比表面积和良好的稳定性,对替考拉宁具有显著的吸附性能,为在水溶液中去除含大分子顺式二醇化合物提供了广阔的前景。由于UiO-66具有优越的稳定性和相对耐水、耐酸性等特点,研究人员也通常通过预安装策略将其用来构建硼酸功能化的MOFs^[47,48]^。

**图5:**
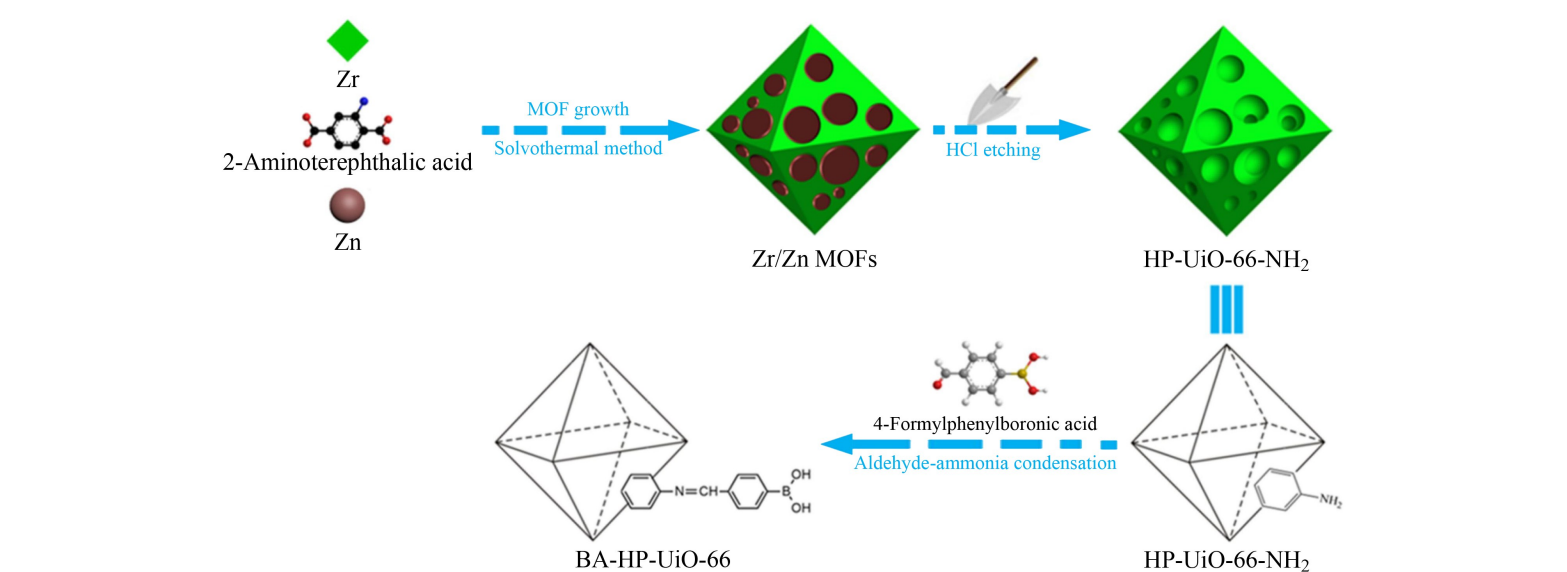
BA-HP-UIO-66的合成示意图^[46]^

基质辅助激光解吸电离飞行时间质谱(MALDI-TOF-MS)是近年来发展起来的一种新型的软电离生物质谱,是一种强有力的分析测试手段,被广泛应用于分析生物大分子,并且表现出了许多优点,如样品制备简单、盐耐受性好、速度快、灵敏度高、可检测质量范围广等^[49]^。MALDI-TOF质谱检测具有这些独特优势,能被广泛应用于蛋白质、核酸聚合物和小分子分析等领域,其作用原理是将激光以脉冲的形式照射样品分子,导致样品的解吸和电离,然后用TOF-MS检测样品。MALDI-TOF质谱的激光解吸/电离过程和信号强度通常取决于基质,但其用于小分子化合物的分析仍有一定的局限性。为了克服限制,研究人员研究了许多材料,其中MOFs在UV-vis范围^[50]^中具有较高的摩尔吸收系数,可以被用作小分子化合物MALDI-TOF质谱分析的基质。Li等^[51]^通过水热反应和逐步改性,合成了硼酸功能化的磁性MOFs(Fe_3_O_4_@PDA@B-UiO-66),将所制得的Fe_3_O_4_@PDA@B-UiO-66作为吸附剂和基质,用MALDI-TOF质谱进行葡萄糖的分析。而Bei团队^[52]^用金纳米颗粒包裹MOFs,再后修饰上硼酸功能团,得到一种硼酸功能化MOFs,并用基质辅助激光解吸电离质谱法分析人体尿液、血清和茶饮料中的顺式二醇小分子,促进了MALDI-MS的发展,为用MALDI-MS分析小分子领域做出了重要和积极贡献。

硼酸盐亲和材料因其优良的性能,常被用于识别糖蛋白/糖肽。Saleem等^[53]^在石墨烯片上原位生长UiO-66-NH_2_,再通过后修饰方法制备得到亲水性硼酸功能化的复合纳米粒子(GO@UiO-66-PBA),并将其用于*N*-糖肽的富集分离分析。另外,分子印迹技术是提高材料特异性识别能力的有效方法^[54]^。该技术是将模板印迹在纳米颗粒表面,然后洗脱模板,留下与模板分子形状一致的空腔,获得印迹聚合物^[55⇓⇓-58]^。研究人员通常会将分子印迹技术和硼酸亲和作用相结合,实现对顺式二醇化合物的特异性识别。Wu等^[59]^将TPA、金属有机骨架和分子印迹技术相结合(见图6),开发了一种新型的硼酸功能化纳米材料MIPs/TBA/MOF@Fe_3_O_4_,用于在生理pH条件下对糖蛋白进行分离与浓缩。研究表明,有了TBA和印迹位点的双重识别,MIPs/TBA/MOF@Fe_3_O_4_纳米粒子在生理条件下具有更好的结合性能(337.8 mg/mL)和对糖蛋白的选择性,制备的纳米颗粒在蛋清样品的应用中表现出良好的性能。

**图6:**
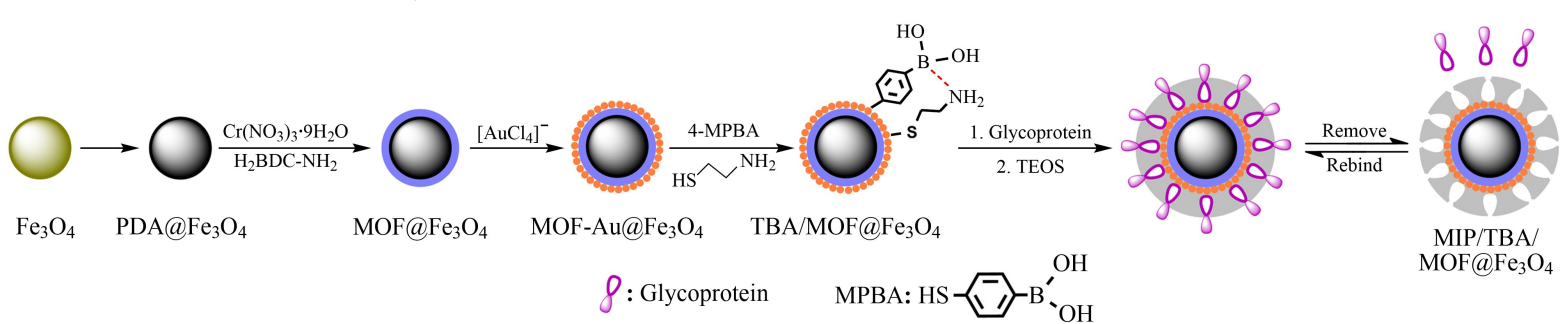
MIPS/TBA/MOF@Fe_3_O_4_的制备流程示意图^[59]^

### 1.2 硼酸功能化MOFs的种类

#### 1.2.1 磁性MOFs

为了能更好地分离实际样品和MOFs,研究人员通常会对MOFs进行修饰,使其磁化,磁性MOFs既能实现固液两相的快速分离,又具有高选择性富集的优良性能。但是,超顺磁纳米粒子的聚集会破坏MOFs的生长。聚多巴胺(PDA)可以在温和条件下自发聚合,降低原粒子的超顺磁性,是一种适合于磁芯涂层的聚合物。此外,由于PDA具有羟基和氨基基团,Zn^2+^很容易黏附在表面,从而产生了硼酸配体逐层合成的方法,并且PDA壳层会赋予Fe_3_O_4_@PDA一定的亲水性。Liu等^[29]^将BA-MOFs修饰在PDA包覆的磁性微球上制备了亲水性磁性硼功能化金属有机骨架(Fe_3_O_4_@PDA@BA-MOFs)纳米复合材料,具有形貌均匀、饱和磁化强度高、磁响应性好等特点,用于选择性富集木犀草素,为制备多功能磁性MOFs材料用于吸附、催化和靶向给药治疗带来了巨大的可扩展性。磁性MOFs的例子还有很多,比如,Xie等^[60]^通过在聚乙烯吡咯烷酮(PVP)和聚乙烯亚胺(PEI)包被的磁性微球上以1,4-苯二硼酸(PBA)和TPA为有机配体改性MOFs(见图7),设计并合成了一种新型硼酸功能化的磁性金属有机骨架(Fe_3_O_4_@PVP/PEI@MOF(B)),对糖肽进行了高效选择性富集。Zhang等^[61]^通过3-羧基苯基硼酸预安装策略,成功制备了一种新型硼酸功能化磁性MOF复合物(MNPs@Zr-MOFs-BA)选择性富集分离核苷。

**图7:**
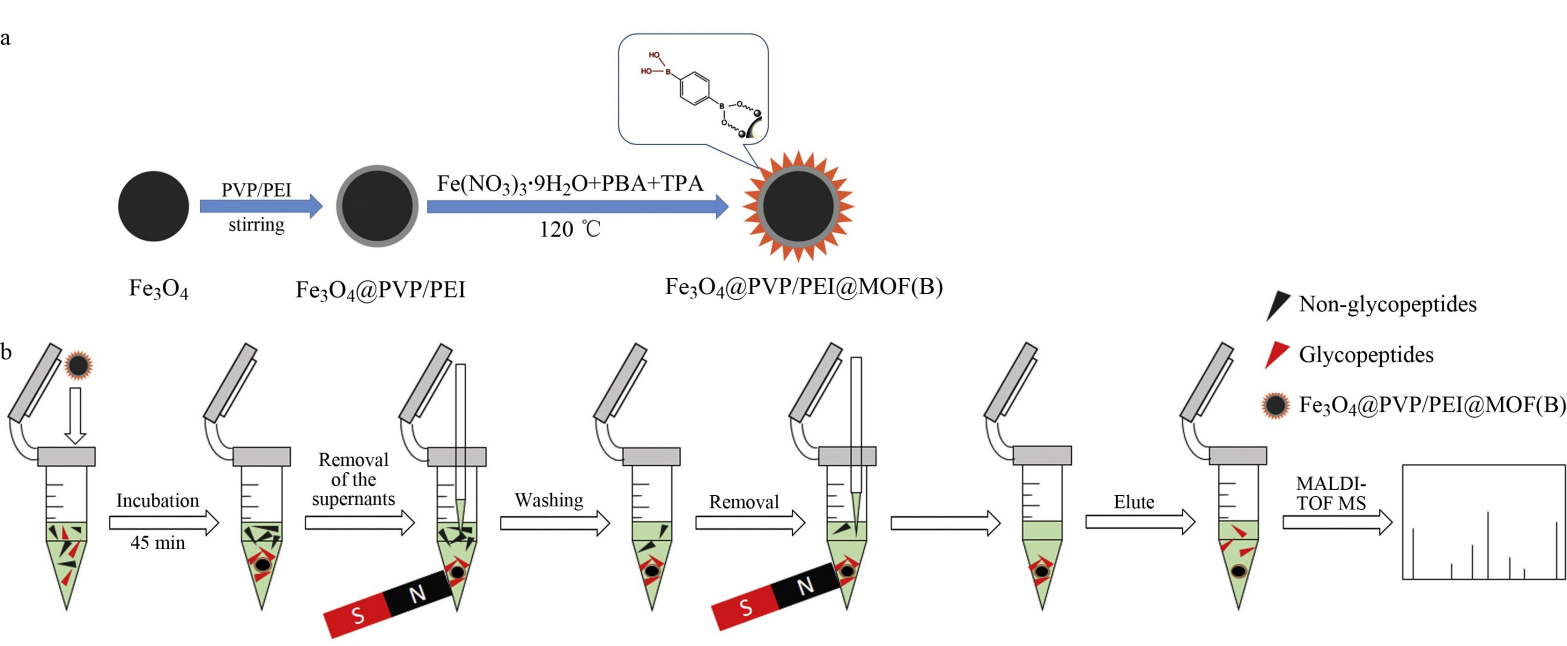
(a)Fe_3_O_4_@PVP/PEI@MOF的合成路线及其(b)从生物样品中富集糖肽的工作流程图^[60]^

在合成MOFs的过程里,中间层的合成是复杂和耗时的,而且一般中间层的原材料要么昂贵,要么对人类有毒。研究人员发现单宁酸(TA)作为天然丰富的多酚类物质之一,价格便宜、安全、对环境友好,而且在温和的条件下,TA可以在数分钟内与不同的金属离子配位,在各种底物上形成单宁酸金属聚合物(PTA)^[62,63]^。由此可得,PTA层可能是硼酸功能化MOFs负载到磁性纳米粒子(MNPs)上的一个很好的基底材料。Xing等^[64]^使用PTA中间层包覆Fe_3_O_4_颗粒,采用MLFC方法将MIL100(Fe)-B修饰在PTA包覆的Fe_3_O_4_上(见图8),制备了亲水性磁性硼酸功能化金属-有机骨架(Fe_3_O_4_@PTA@MIL-100(Fe)-B)纳米复合材料,并用于选择性富集人类尿液样本中的儿茶酚胺(Cas),为制备多功能MOFs磁性材料应用于临床研究和治疗提供一种研究思路。

**图8:**
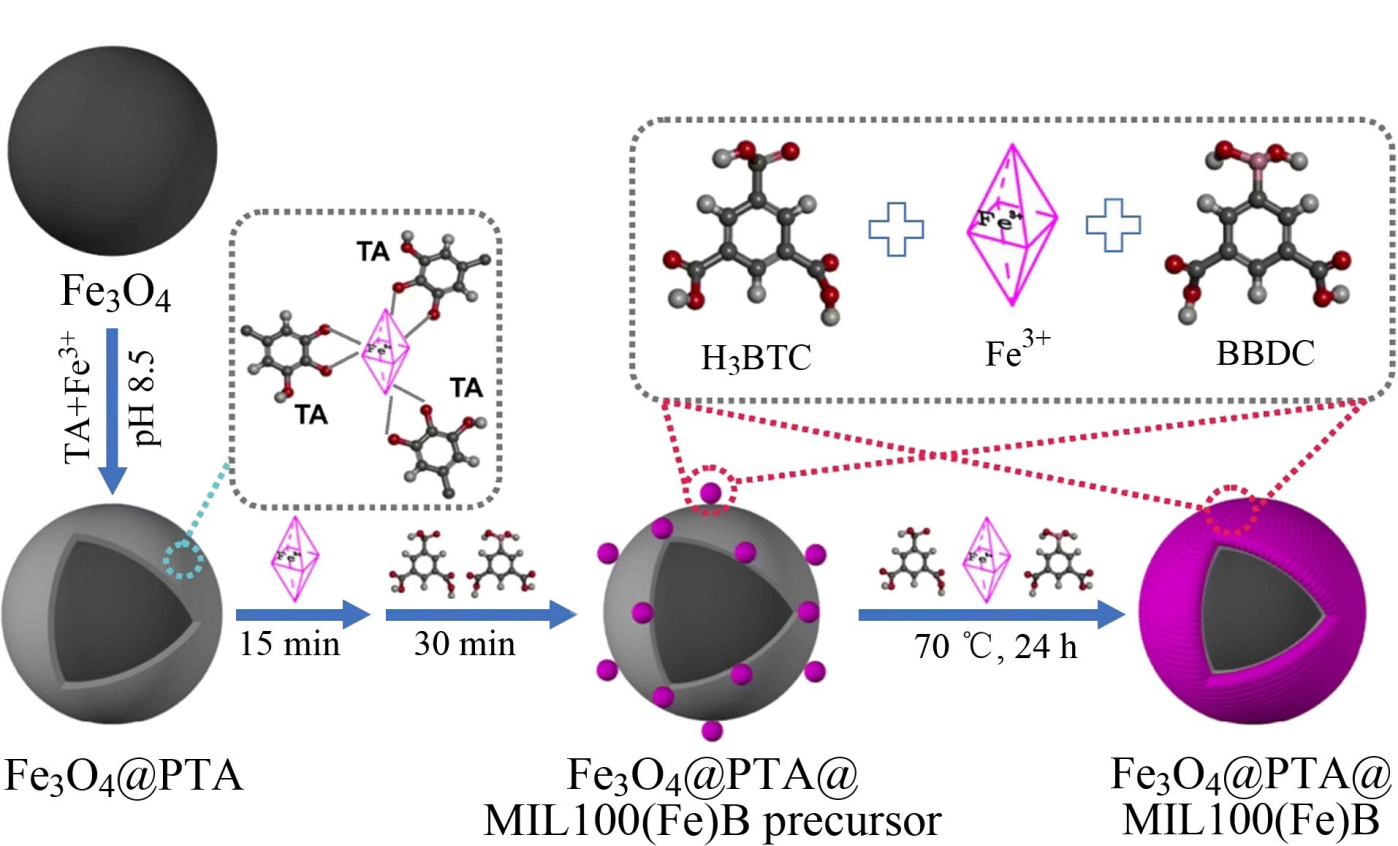
Fe_3_O_4_@PTA@MIL-100(Fe)-B的设计合成示意图^[64]^

#### 1.2.2 硼酸功能化Ln-MOFs

在种类繁多的MOFs中,镧系MOFs(Ln-MOFs)由于具有较大的斯托克斯频移、较高的量子产率、较长的衰变寿命和不受干扰的发射能量等优良的发光特性,备受关注。在Ln-MOFs中,由于配体向Ln离子的能量转移,配体的芳香发色团能够吸收紫外线激发Ln离子^[65]^,然而,当芳香配体被官能团修饰后,在特定的发射光谱下,能量转移效率会发生变化。因此,调节能隙的关键是选择具有理想官能团的有机配体,而硼酸基团是缺电子的,可以调节配体的能级,获得具有独特荧光效率的MOFs。Zhang等^[66]^利用Ln-MOFs优异的发光性能,及硼酸对二醇化合物亲和特异性结合性质,制作了一种可以检测尿路肺癌生物标志物*N*-乙酰神经氨酸(NANA)的分子机器人以及NANA传感系统,实现对早期肺癌的检测和预防。在该NANA传感系统中,硼酸基团作为驱动器,利用“功能手”抓取NANA的分子机器人。B-EuMOF的荧光( ${I_{470}}_{nm}$ / ${I_{614}}_{nm}$ )作为分子机器人的传感器,其功能是检测来自其周围环境的信息。与其他分子机器人相比,该荧光传感器显示的传感结果肉眼可见,这使得传感结果更加直观和非专业,为荧光传感在人工智能早期疾病监测领域的应用提供了新的可能性,也为拓宽分子机器人的应用领域铺平了新的道路。Du等^[67]^以3,5-二羧基苯硼酸(DBA)为功能配体,以Eu^3+^离子为金属节点,制备了Eu-MOFs(见图9),并引入硼酸基团,合成出Eu-MOF比率探针。将其作用于多巴胺(DA)后,该材料表现出良好的分散性和高选择性,同时具有较宽的响应范围和较低的检出限(0.015 μm),该材料也可用于其他顺式二醇化合物的检测,在化学和生物传感领域具有广阔的应用前景。

**图9:**
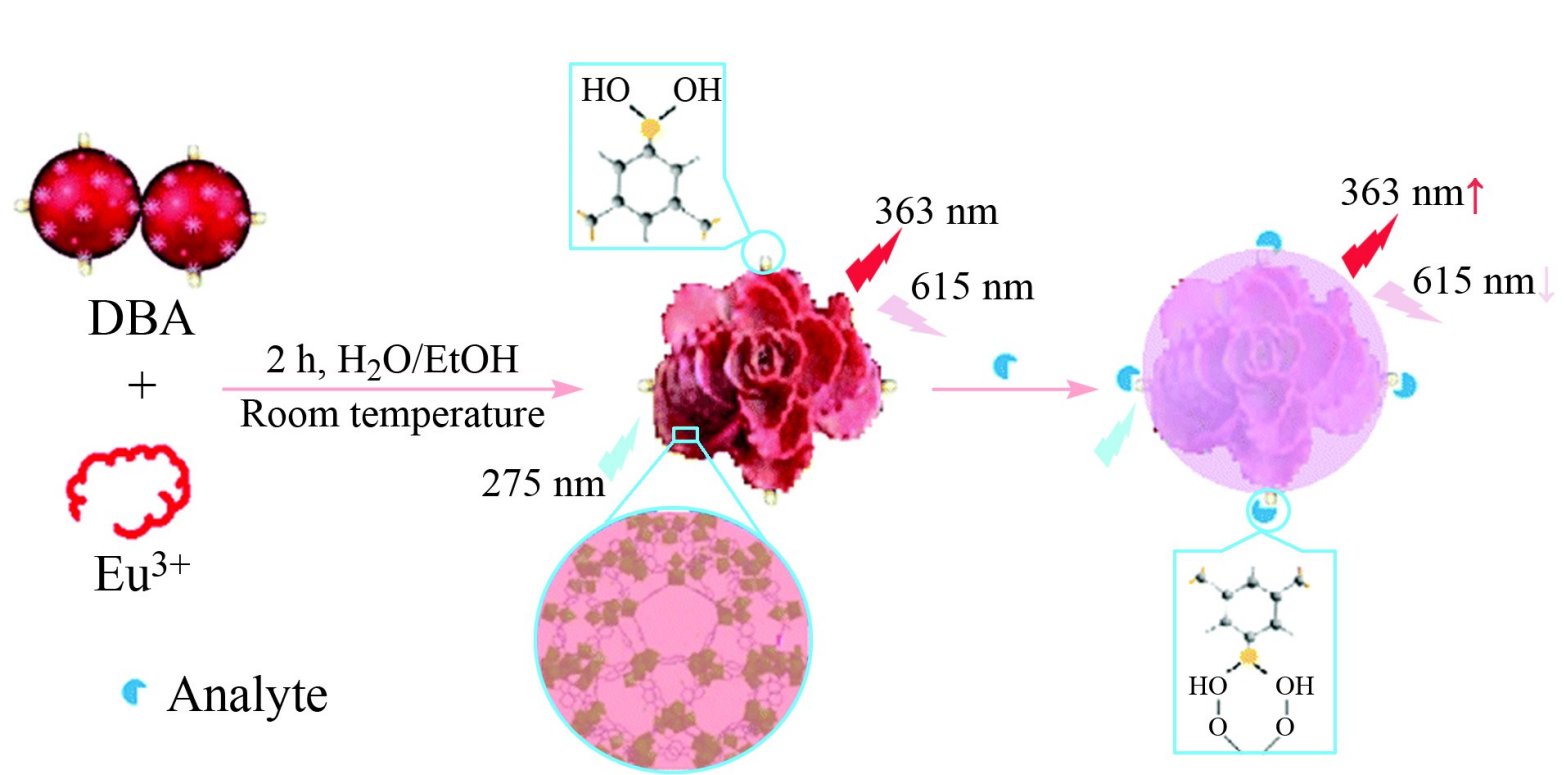
Eu-MOF探测系统的合成过程示意图^[67]^

#### 1.2.3 硼酸功能化MOF膜复合材料

近年来在分离领域,MOF膜复合材料与传统分离工艺中未成型MOF材料相比,具有能耗低、泄漏污染小、分离效率高等优点,在分离领域受到了广泛关注^[68⇓-70]^。优化后的MOF膜复合材料具有较高的吸附容量、良好的机械强度和化学稳定性,特别是表面性能可调控。同时,研究表明,基于疏水多孔基质制备耐水MOF膜复合材料将大大提高其在实际含水样品(水相条件下)中的分离能力^[71]^。综上所述,Liu等^[72]^利用Zn(Ⅱ)-配体-片段共组装和界面诱导相结合的方法,在疏水多孔类泡沫碳衬底上制备苯硼酸功能化MOF膜(BA-MOF-PFCS)(见图10),用于在水相条件下选择性分离天然药物木犀草素。实验发现,BA-MOF-PFCS对LTL选择性的提高不仅仅是邻苯二酚与苯硼酸之间的特异性相互作用,还有“分子筛效应”的协同作用。在含水溶液中,BA-MOF-PFCS复合材料可以轻松提取纯度为85%的LTL,并将其浓缩到99.90%,该研究为制备膜状硼酸功能化MOF复合材料提供鲜明的思路。

**图10:**
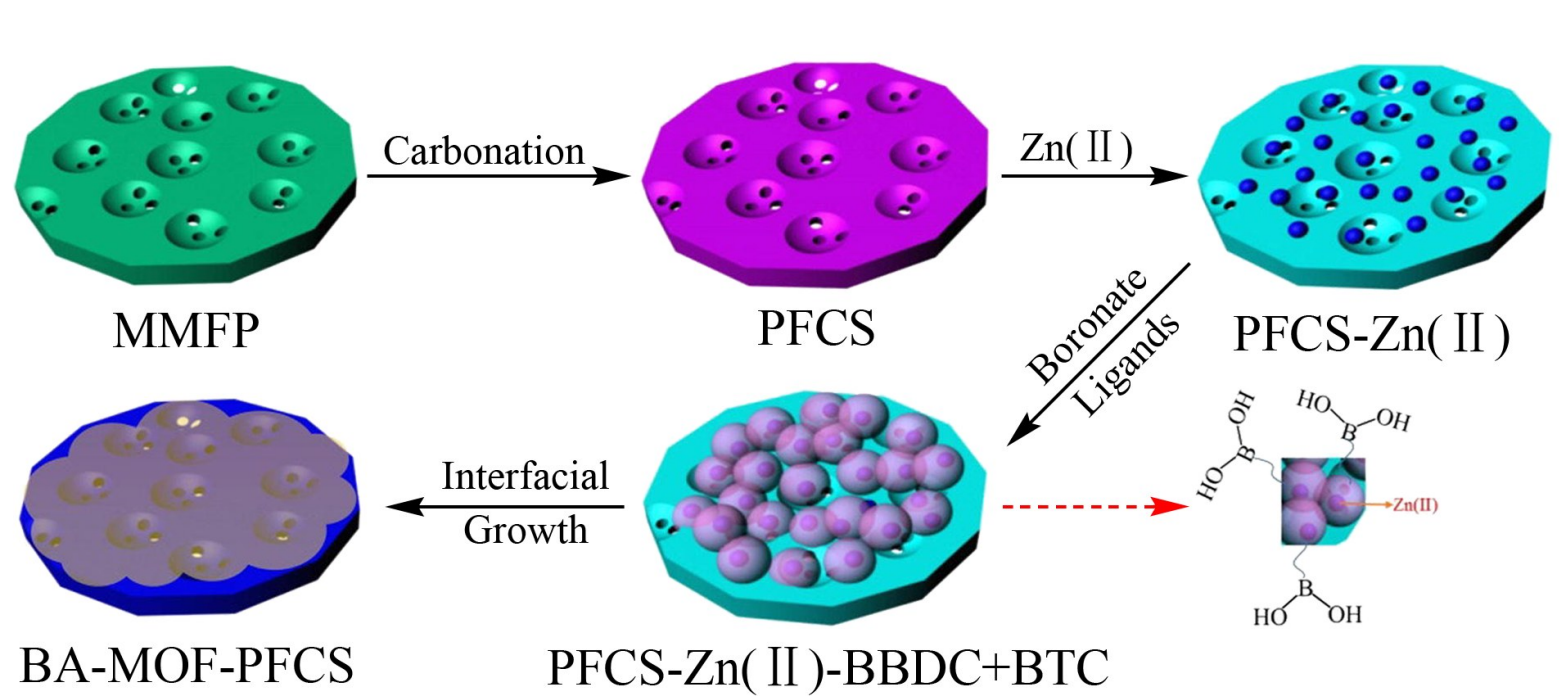
界面诱导锌(Ⅱ)-配体-片段共组装策略在疏水性多孔泡沫碳基膜(BA-MOF-PFCs)上制备硼酸盐基MOF膜^[72]^

## 2 硼酸功能化COFs

COFs作为有机结晶多孔材料^[73,74]^,自2005年Yaghi等^[75]^首次报道以来,因其低质量密度、规则的孔隙结构、高比表面积、易于改性等特点,已成为吸附、催化剂、分离和能源利用等领域研究的热点。COFs遵循网状化学原理,其结构与MOFs相似^[76]^,与MOFs相比,该材料具有显著的优点,包括更好的化学稳定性和*π*-*π*堆积相互作用^[77]^,这些优点使得COFs在分离科学领域的研究越来越受到重视。

目前制备功能化COFs的方法主要有两种,一种是“自下而上”的策略,也可称为配体共组装法,在这个方法中,功能性的建筑单元直接作为建筑块来构造功能化的COFs材料,且材料中的官能团具有良好的均匀性,然而,“自下而上”的策略要求建筑单元的结构具有优良的对称性和稳定性,同时COFs合成的恶劣条件可能会破坏功能群^[32]^,使得合并的功能群非常有限。另外,当不同的官能团连接到相同的父系结构构造建筑单元时,相应的COFs的合成条件可能存在显著的差异,这使得“自下而上”的策略不具备通用性。另一种功能化策略是“合成后修饰”策略,其优点在于可以根据特定功能化的需要,直接将不同功能的基团引入材料中,但是这种策略存在着改变晶体结构的风险。

硼酸功能化的COFs具有较大的比表面积、硼酸反应点和超强的稳定性,也具有选择性富集能力和优良的广泛适用性。与传统COFs相比,硼酸功能化的COFs对顺式二醇化合物具有更高的选择性和灵敏度。目前,硼酸功能化COFs在色谱分离和样品前处理领域应用不多。现就报道的硼酸功能化COFs的制备及其应用于顺式二醇类化合物分析做一个归纳总结。

### 2.1 “自下而上”策略

Hu等^[78]^以2,4,6-三羟基-1,3,5-苯三醛、联苯胺和4-氨基苯基-4-硼酸为配体,采用“一锅法”,合成了硼酸功能化COFs(B-COFs),用于选择性吸附顺式二醇化合物(木犀草素、核黄素和邻苯二酚),在无干扰背景下直接用于MALDI-TOF质谱检测。研究结果表明,合成出的B-COFs与其他传统基质相比具有巨大优势,如在不含三氟乙酸的低质量范围MS中无干扰背景等。该策略为其他功能化COFs的合成以及其在复杂样品中富集靶分子提供了一定的理论支撑。然而,所报道的B-COFs的比表面积(238 m^2^/g)和孔体积(0.13 cm^3^/g)不高;同时,亚氨基复合材料由于具有热力学不稳定的性质,限制了其在应用上的可逆性。因此,研究稳定的COFs越来越受到人们的关注。Liu等^[79]^利用aza-Diels-Alder环加成反应将亚胺型COFs转化为喹啉型COFs,通过形成芳香环骨架进行动力学固定,不仅提高了COFs的稳定性,而且实现了COFs的后修饰。

### 2.2 “合成后修饰”策略

Chang等^[80]^以TPB-DMTP-COF(TPB:三苯基苯;DMTP:二甲氧基对苯二甲醛)为前体,通过aza-Diels-Alder环加成反应,成功设计并制备了硼酸功能化共价有机骨架(COF-BA)(见图11),研究发现COF-BA对1,2-二羟基蒽-9,10-二酮(1,2-Doa)具有独特的吸附选择性,而对其异构体1,4-二羟基蒽-9,10-二酮和2,6-二羟基蒽-9,10-二酮的吸附效果较差。COF-BA具有持久的孔结构、高比表面积(606 m^2^/g)和均匀的孔径(2.59 nm),并且COF-BA具有本征荧光性质,在365 nm紫外光照射下,COF-BA呈蓝色荧光,而1,2-Doa/COF-BA呈橙色荧光(见图12),利用这一特性,此材料可以用作1,2-Doa的光学传感器,在吸附和传感领域具有巨大的潜力和广阔的应用前景。

**图11:**
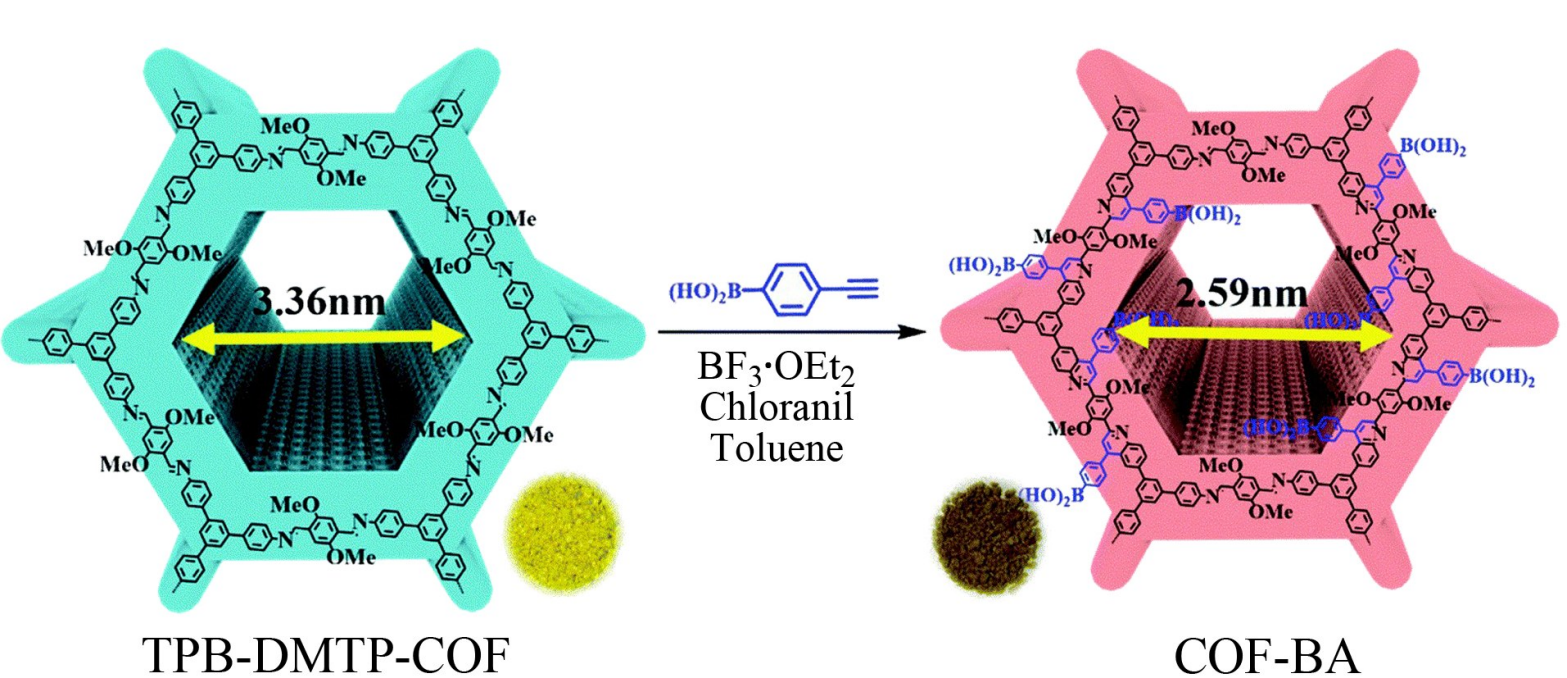
由TPB-DMTP-COF合成共价有机骨架COF-BA的合成路线^[80]^

**图12:**
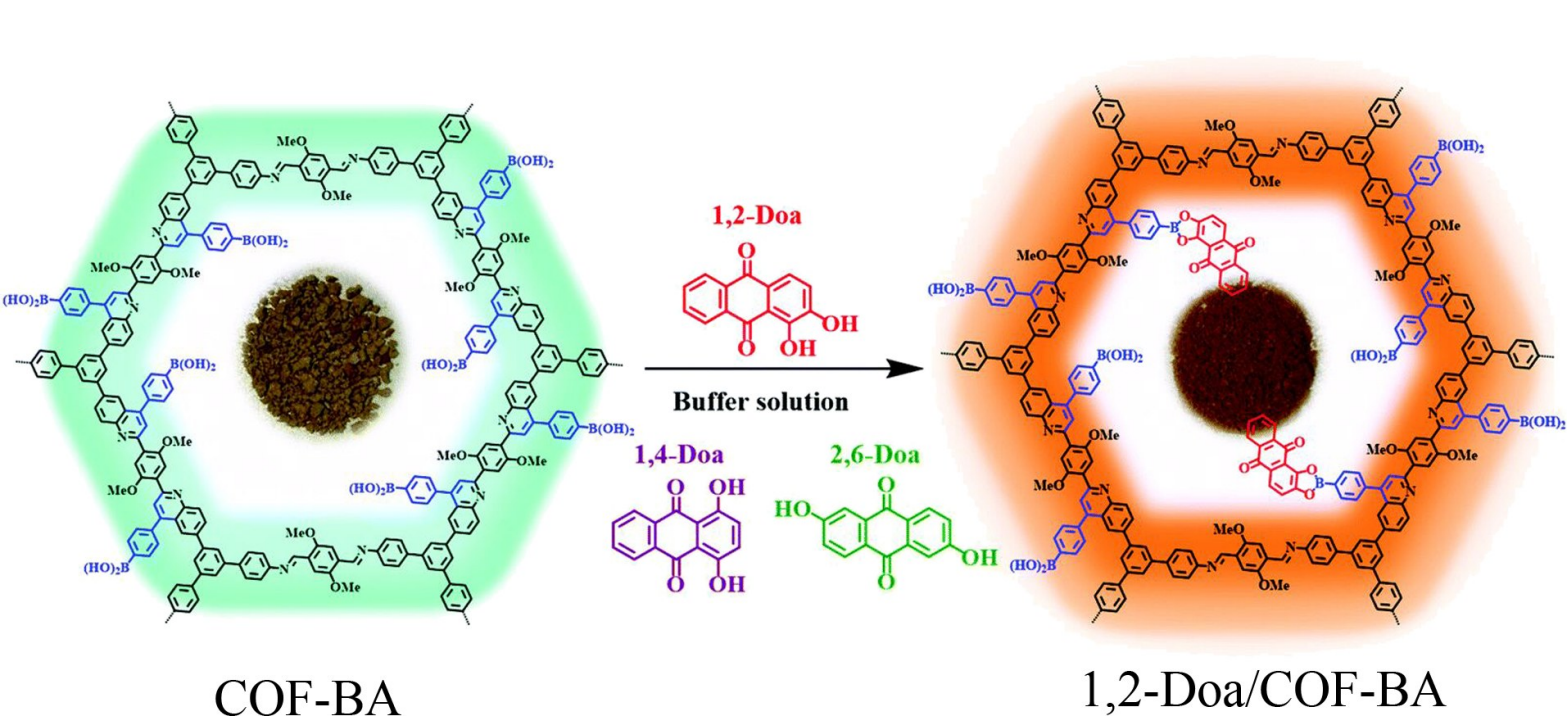
共价有机骨架COF-BA对1,2-Doa的特异性吸附过程^[80]^

硫醇-烯点击法具有高效、高选择性等优点,是合成功能化COFs的一种可行方法。如图13所示,Ji等^[81]^以含乙烯基的2,5-二烯丙基氧基对苯二甲酸(Da-V)和1,3,5-三(4-氨基苯基)苯(Tab)为配体,制备了含乙烯基的COF DhaTab-V,并用DhaTab-V与4-巯基苯硼酸(4-MPBA)进行巯基烯点击反应,得到苯硼酸功能化的COF DhaTab-PBA,用于吸附水介质中邻苯二酚。

**图13:**
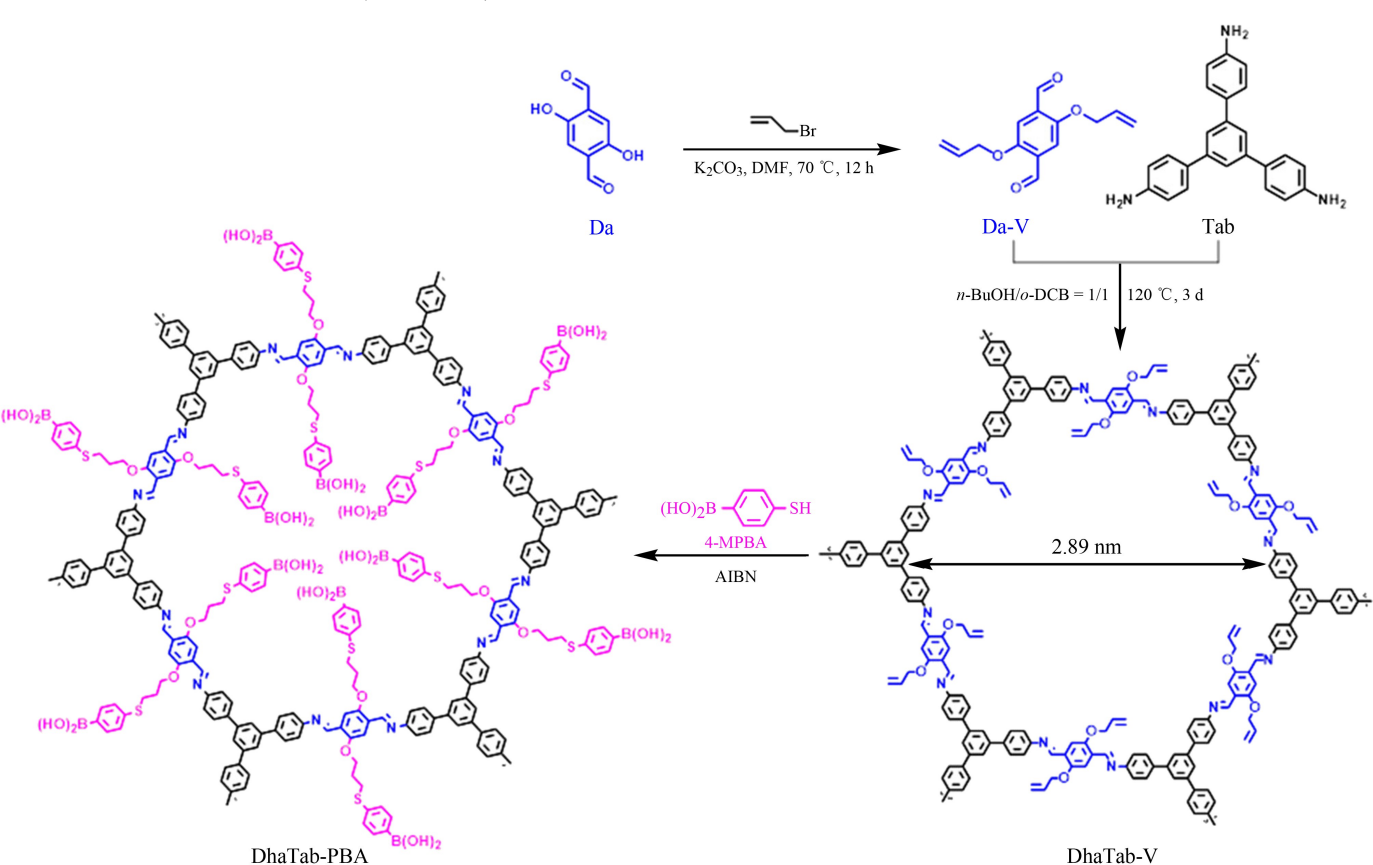
通过Tab和Da-V缩合制备DhaTab-V和通过硫醇-烯点击反应制备DhaTab-PBA的示意图^[81]^

Bein等^[82]^提出了一种新的通过合成后还原反应合成含胺基的*β*-酮烯胺COF的方法。他们先合成了化学稳定的*β*-酮烯胺COF TpBD(NO_2_)_2_,并在孔隙中锚定了硝基,之后将硝基还原,得到了所需材料COF TpBD(NH_2_)_2_。受此启发,Wang等^[83]^制备了含氨基核-壳结构的磁性COF纳米球[Fe_3_O_4_@TpBD(NH_2_)_2_],其中氨基基团作为进一步硼酸功能修饰的活性位点,最后制得Fe_3_O_4_@COF@2-FPBA纳米颗粒,并将其应用于高选择性萃取单胺神经递质(MNTs)。

COFs相对于MOFs来说具有更好的化学稳定性^[84]^,被广泛应用于糖蛋白/糖肽类化合物的MALDI-TOF质谱分析。Gao等^[85]^以磁石胶体纳米晶团簇(MCNCs)为核,苯硼酸修饰的COFs为壳,通过叠氮化-炔“点击”反应合成一种具有吸引力的核壳结构的苯硼酸固定磁性共价有机框架(MCNCs@COF@PBA),并将其用于*N*-糖肽的特异性富集(见图14)。由其应用于分析HeLa细胞分泌的外泌体中的糖肽的结果来看,此材料的选择性、灵敏度、富集回收率和磁选方面都比较优异,因此,研究不仅为糖蛋白组综合谱分析提供了一个潜在的选择性富集平台,也为COFs基材料的通用功能化开辟了新的途径。

**图14:**
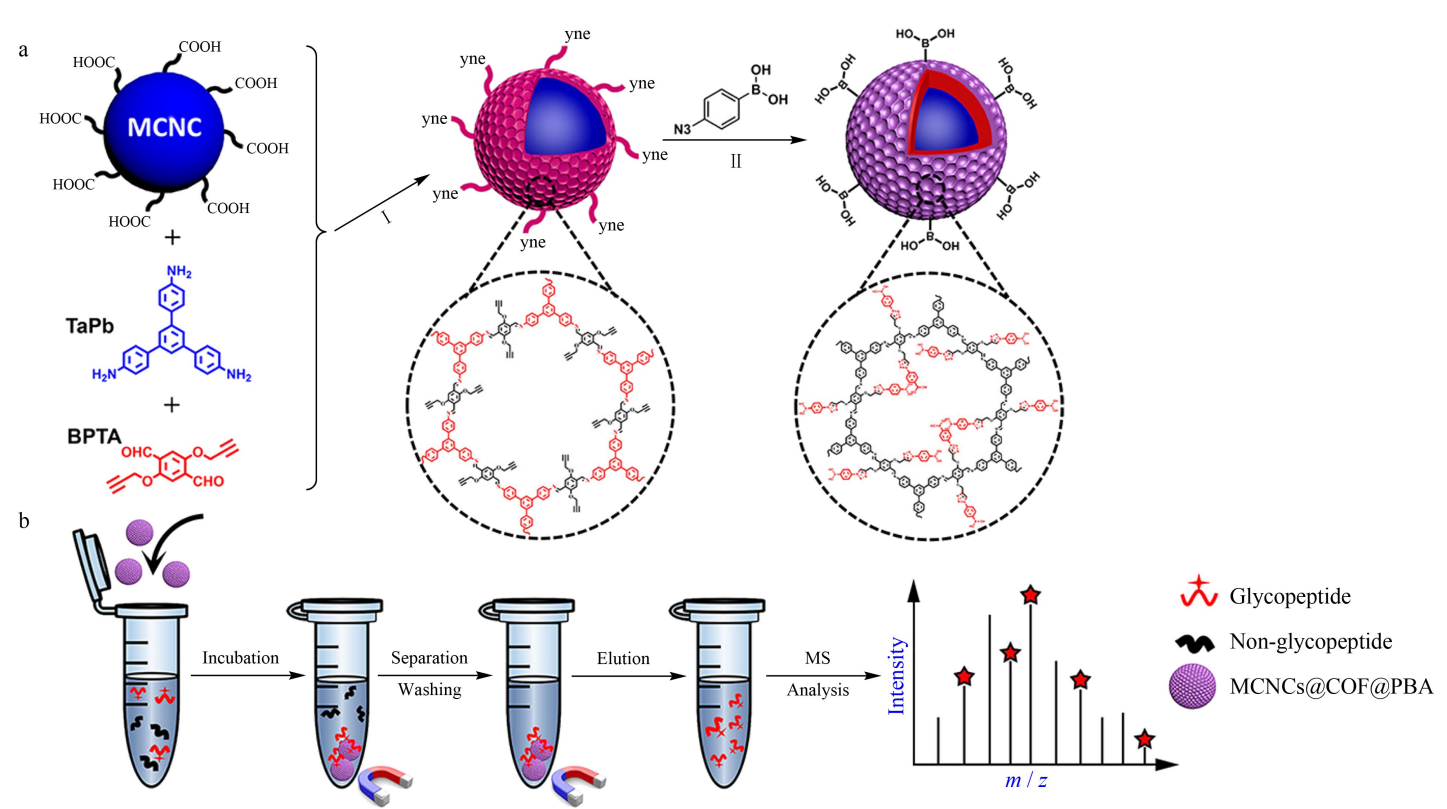
(a) MCNCs@COF@PBA复合材料合成过程示意图和(b)磁性可回收肽分离过程示意图^[85]^

Xie等^[86]^通过开环反应对COFs表面的丁二酸酐(SA)进行改性,然后通过静电作用将带正电荷的聚乙烯亚胺(PEI)修饰在COFs上,使PEI作为还原剂和固定剂进一步固定金纳米粒子。最后,通过Au纳米颗粒与巯基的反应,使4-巯基苯硼酸(4-MPBA)成功修饰到材料表面,得到了硼酸功能化的TbBD@PEI@Au@4-MPBA材料(见图15),并将其应用于对糖肽的选择性富集。

**图15:**
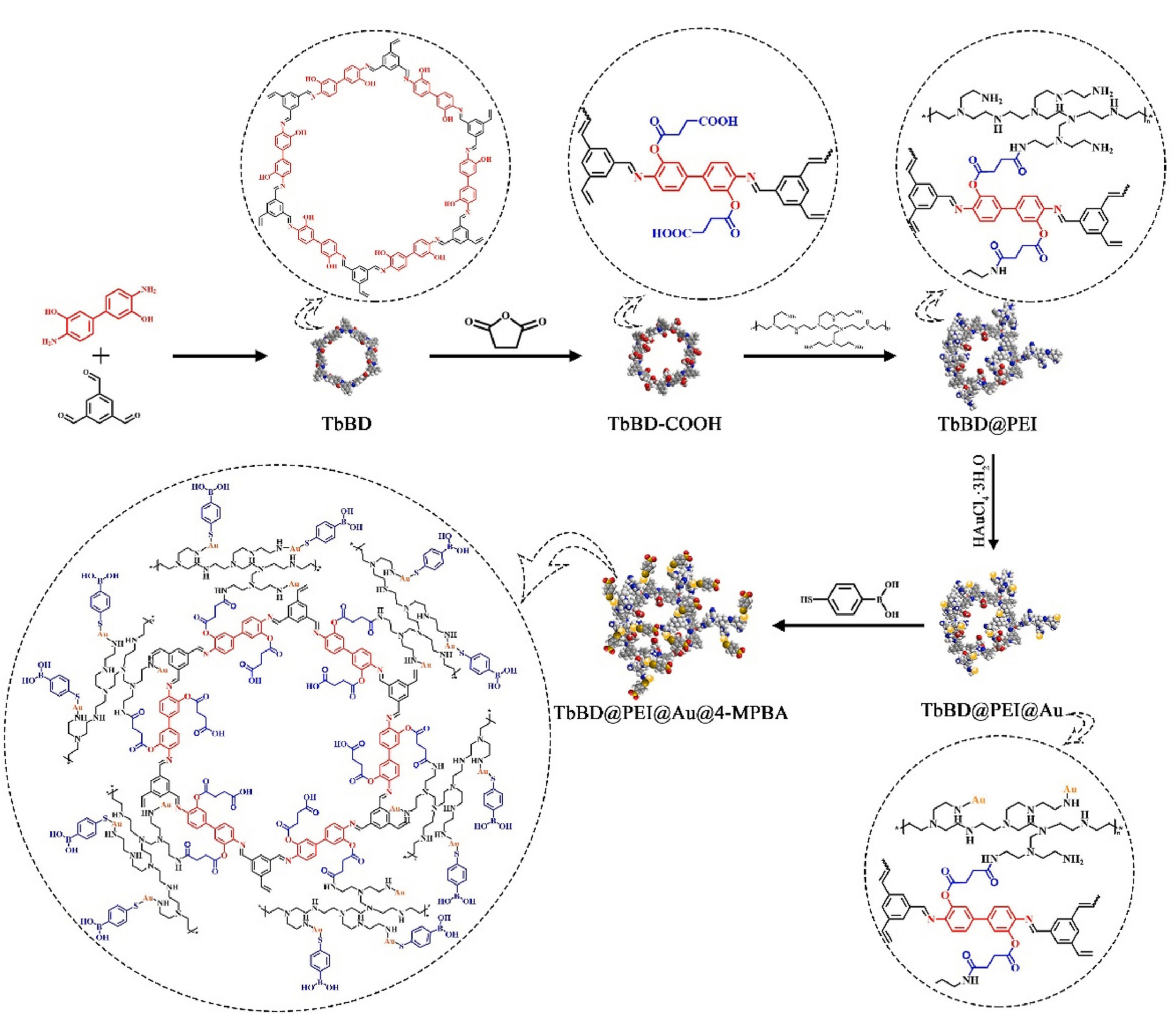
TbBD@PEI@Au@4-MPBA复合材料的合成示意图^[86]^

研究结果表明,TbBD@PEI@Au@4-MPBA不仅表现出对糖肽类物质显著的富集选择性(当糖肽物质辣根过氧化物酶胰酶与非糖肽干扰物质牛血清白蛋白胰酶的质量比为1:1000时,仍能选择性检测到辣根过氧化物酶胰酶),而且具有较高的灵敏度(5 amol/μL),且该材料的重复性和稳定性测定结果也较好。最重要的是,在实际样品检测中,MALDI-TOF MS检测到从新鲜人唾液中富集的56种内源性糖肽,用纳米LC-MS/MS鉴定了56种对应于31种人唾液胰蛋白酶消化液糖蛋白的内源性糖肽,用纳米LC-MS/MS鉴定了513种对应于208种喉癌患者血清胰蛋白酶消化液糖蛋白的糖肽。优异的研究结果表明,改性后的新型COFs材料有望用于从各种复杂的生物样品中富集糖肽,并且该材料的成功合成为COFs在蛋白质组学中的应用提供了新的思路。

## 3 结论与展望

随着多孔材料不断被开发和制备,硼酸修饰的MOFs和COFs的种类也逐渐增多,其对各种顺式二醇化合物富集选择性方面的应用也随之增多,且有着巨大的进步。在硼酸修饰MOFs的制备方法方面,大部分将硼酸官能团引入MOFs的方法是MLFC策略,与其他方法相比,提高了MOFs的吸附和富集效率。另外,MOFs本身的一些缺点如聚集问题、孔径问题等也得到很好的解决。同时,研究人员也会根据顺式二醇化合物的自身特点来设计出性能优良的硼酸亲和MOFs材料。并且,为了方便富集分离,许多研究者会将硼酸功能化MOFs制成磁性材料。此外,Ln-MOFs的发光特性也被利用,与硼酸亲和作用相结合,用于一些顺式二醇化合物的富集分离和检测。而在COFs方面,“合成后修饰”策略和“自下而上”策略是制备硼酸功能化COFs的两种主要方法,硼酸功能化COFs的例子相对MOFs而言是比较少,其应用价值还有待继续挖掘。总的来说,目前硼酸亲和MOFs和COFs在生物、医疗检测和工业方面都有着巨大的应用价值,并且在经过了十几年的发展后,各项研究也有了突破性的进步,相信在不久的将来,硼酸亲和MOFs和COFs材料会在实际应用方面尤其是蛋白质组学和代谢组学的研究中发挥巨大作用。
